# Breast reconstruction in pregnancy: a case report of multidisciplinary team approach in immediate autologous flap reconstruction for pregnancy‐associated breast cancer

**DOI:** 10.1002/ccr3.1092

**Published:** 2017-07-20

**Authors:** Prakasit Chirappapha, Panya Thaweepworadej, Nuttapong Ngamphaiboon, Matchuporn Sukprasert, Thongchai Sukarayothin, Monchai Leesombatpaiboon

**Affiliations:** ^1^ Department of Surgery Faculty of Medicine Ramathibodi Hospital Mahidol University Bangkok Thailand; ^2^ Department of Medicine Faculty of Medicine Ramathibodi Hospital Mahidol University Bangkok Thailand; ^3^ Department of Obstetrics & Gynecology Faculty of Medicine Ramathibodi Hospital Mahidol University Bangkok Thailand

**Keywords:** Immediate autologous flap reconstruction, pregnancy‐associated breast cancer, transverse rectus abdominis musculocutaneous flap

## Abstract

This report presents the results of immediate breast reconstruction with autologous flap in Pregnancy‐associated breast cancer (PABC). There was no obstetrics and surgical complications in our report. Immediate breast reconstruction can be performed in PABC after a careful selection. Multidisciplinary team approach is the key in managing these groups of patients.

## Introduction

Pregnancy‐associated breast cancer (PABC) is generally defined as cancer diagnosed during pregnancy and the first year after delivery. Earlier reports have shown incidences of PABC varying from 1 in 10,000 to 1 in 3000 deliveries 1, 2, 3, 4. Although the incidence was uncommon, it was the most common cancer affecting pregnancy. Management of PABC is complex and involves many aspects, including patient, family, and the medical team. There are two individuals concerned, the mother and her child. Patients with PABC often present with advanced stage. However, previous studies reported no difference in prognosis and survival compared with nonpregnant women by stage 5, 6. The treatment modalities are based on the trimester of pregnancy, choosing appropriate treatments with less toxicity to the fetus. Multidisciplinary team (MDT) approach is an important key in managing this group of patients. As radiotherapy is contraindicated in pregnancy, mastectomy is often unavoidable. Most of patients with PABC affect young women 1. This makes the cosmetic outcome one of the most concerns. Breast reconstruction is usually preferred by patients. Most surgeons choose to delay reconstruction to minimize the surgical risk in pregnant women at the expense of cosmetic result. Immediate reconstruction is rarely performed. To date, there are few case series of immediate reconstruction in PABC reported in the literature.

Two case series performed immediate reconstruction in PABC had been reported (Table 1). Lohsiriwat et al. reported a series of 13 patients with breast cancer who had immediate reconstruction during pregnancy. Most patients underwent a two‐stage procedure with expander 7. Only one patient had definitive implant. No major complications or fetal malformations were observed in this study.

**Table 1:** Case series of immediate breast reconstruction in PABC

Author	Year	No. cases	GA (week)	Operative technique	Complication
Lohsiriwat et al. 7	2013	13	7–26	12 cases with tissue expanders one case with definite implant	None
Caragacianu et al. 8	2016	10	7–30	Tissue expanders	Skin flap necrosis one case

Caragacianu et al. presented safety and favorable outcomes after performing immediate reconstruction with tissue expanders in 10 patients with PABC 8. Operative time was slightly longer in patients who underwent immediate reconstruction, which was not compromised obstetric and fetal outcomes. One patient had intraoperative premature uterine contraction during operation and treated with tocolysis.

## Case Presentation

**Figure 1:**
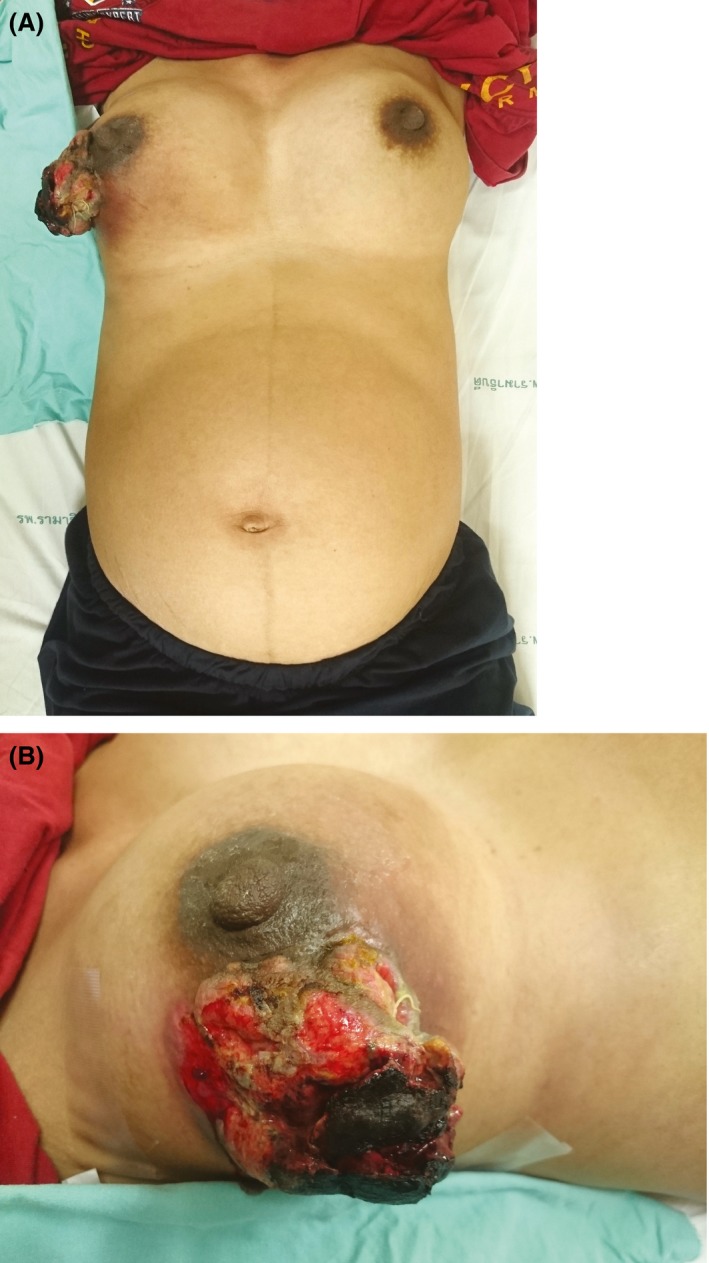
(A) Anterior view of huge ulcerated mass at right breast with 32 weeks pregnant. (B) Lateral view of a bulging mass apparent on inspection.

A 35‐year‐old female patient who was 32 weeks pregnant presented with a right breast mass for 7 months. The physical examinations showed a huge ulcerated mass on her right breast and several large right axillary lymph nodes (Fig. 1). The pathology report showed triple‐negative poorly differentiated invasive ductal carcinoma. Her Ki67 score was 90%. Limited metastatic work‐up was completed. She was diagnosed with a locally advanced‐stage cancer (cT4bN0M0). She was multipara with prior four live births by normal deliveries with no previous medical history. She had breast augmentation with subpectoral implants 8 years ago. Her prenatal examination was normal. After discussing with the patient and her family, she decided to continue pregnancy. The treatment plan was discussed in the MDT meeting and her family.

**Figure 2:**
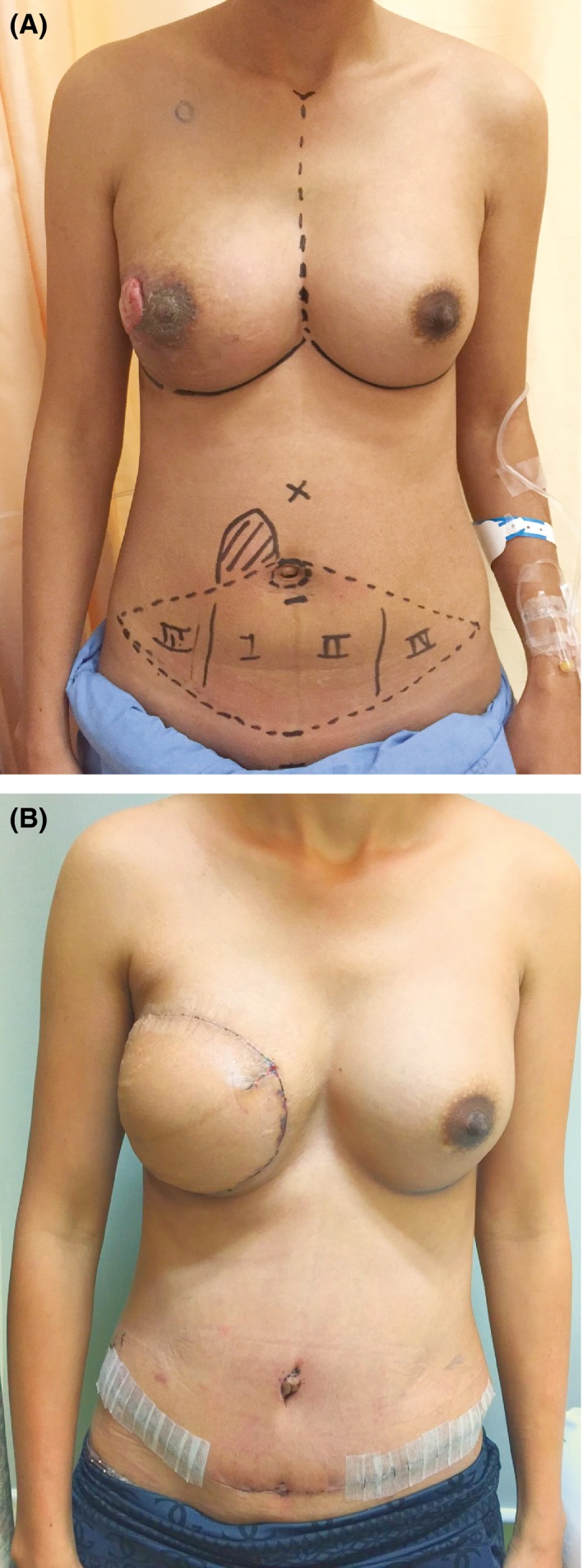
(A) The tumor has shrunk after receiving preoperative chemotherapy. (B) Postoperative view after performing a pedicled TRAM flap with implant.

Neoadjuvant chemotherapy was initiated. After one cycle of doxorubicin and cyclophosphamide (AC), she had slight tumor shrinkage. The obstetrician induced labor with prostaglandin after 36 weeks of gestation by a vaginal delivery without complications. The baby was healthy and weighted 2270 g. After delivery, she continued neoadjuvant chemotherapy with another three cycles of AC, followed by weekly paclitaxel for 3 weeks. Unfortunately, the tumor progressed after the third cycle of paclitaxel. Carboplatin was added to paclitaxel. The tumor remained stable until completion of chemotherapy. Metastatic work‐up showed no distant metastasis. After completion of neoadjuvant chemotherapy, the tumor remained large and required a flap coverage after resection. (Fig. 2A). After discussion with the MDT, she underwent right mastectomy with axillary lymph nodes dissection and immediate autologous reconstruction by transverse rectus abdominis musculocutaneous (TRAM) flap at 20 weeks postpartum (Fig. 2B). Pathology report showed residual invasive ductal carcinoma size 5.5 cm. All resection margins were free from tumor (the nearest part was 0.3 cm from deep resection margin), and the axillary lymph nodes were positive for tumor cells in 18 of 39 lymph nodes (ypT4bN3M0). The tumor was triple‐negative breast cancer by immunohistochemistry staining. She was admitted for 7 days in the hospital without any complications. The patient received postoperative radiotherapy for totally 50.4 Gy in 25 fractions. Unfortunately, the patient was loss to follow‐up after completion of radiotherapy.

## Discussion

Due to rarity, there is no standard treatment in PABC. In our institution, we have four cases of patients with PABC who had surgical operations between 2012 and 2016. The results are demonstrated in Table 2.

**Table 2:** Another three patients performed surgery between 2012 and 2016

Case	Age (year)	GA (week)	Surgery	Axilla	pT	pN	ER/PR	HER‐2	CMT	Relapse
1	33	7	Mastectomy	ALND	2	0	−/+	−	Patient refused	No
2	29	21	Mastectomy	ALND	4	1	−/−	−	Pre‐op	No
3	40	15	Mastectomy	SLNB	2	0	+/+	−	Post‐op	No

GA, gestational age; pT, pathological tumor staging; pN, pathological nodal status; ER/PR, estrogen/progesterone receptor; CMT, chemotherapy; SLNB, sentinel lymph node biopsy; ALND, axillary lymph node dissection.

Managements in PABC could be problematic, not only from obstetrics concerns, but also alteration in breast anatomy by pregnancy. In pregnant women, breast weight can be doubled, more ptosis, and changes in size and pigmentation of nipple areolar complex. These peripartum changes are usually unpredictable, and make more difficulties for decision‐making in surgical techniques. This could be a reason that most surgeons try to avoid an immediate reconstruction technique (especially autologous flap). Previous studies reported the safety and comparable adverse outcomes with nonreconstructed patients. However, most patients had tissue expander and required a second operation.

Our report has shown a result of immediate reconstruction with TRAM flap in patients with PABC. The patient had favorable aesthetic outcomes without postoperative complications and locoregional recurrence. The multidisciplinary approach is a key to manage these complicated patients. To choose an appropriate treatment, surgeons should discuss all possible options with the patient, her family, and MDT team.

## Conclusions

This report presents the results of immediate breast reconstruction with autologous flap in PABC. No obstetrics and surgical complications were observed. Immediate breast reconstruction can be performed in PABC after a careful selection. Multidisciplinary team approach is the key in managing these groups of patients.

## Conflict of Interest

All authors have no financial and personal relationships with other people or organization that could inappropriately influence (bias) their work.

## Authorship

PC: interpreted the data. PT: wrote the manuscript and involved in analysis. NN: critically revised the manuscript. MS: involved in acquisition of data. TS: involved in acquisition of data. ML: involved in acquisition of data.
